# Pulsed thulium laser blood vessel haemostasis as an alternative to bipolar forceps during neurosurgical tumour resection

**DOI:** 10.1007/s10103-023-03747-9

**Published:** 2023-03-27

**Authors:** Alessa Hutfilz, Dirk Theisen-Kunde, Matteo Mario Bonsanto, Ralf Brinkmann

**Affiliations:** 1 Medical Laser Center Lübeck, 23562 Lübeck, Germany; 2 Department of Neurosurgery, University Medical Center Schleswig-Holstein, 23562 Lübeck, Germany; 3 Institute of Biomedical Optics, University of Lübeck, 23562 Lübeck, Germany

**Keywords:** Thulium laser, Haemostasis, Neurosurgery, Blood vessel coagulation

## Abstract

Due to wavelength-specific water absorption, infrared lasers like the thulium laser emitting at 1940 nm wavelength proved to be suitable for coagulation in neurosurgery. Commonly bipolar forceps used for intraoperative haemostasis can cause mechanical and thermal tissue damage, whilst thulium laser can provide a tissue-gentle haemostasis through non-contact coagulation. The aim of this work is a less-damaging blood vessel coagulation by pulsed thulium laser radiation in comparison to standard bipolar forceps haemostasis. Ex vivo porcine blood vessels in brain tissue (0.34 ± 0.20 mm diameter) were irradiated in non-contact with a thulium laser in pulsed mode (1940 nm wavelength, 15 W power, 100–500 ms pulse duration), with a CO_2_ gas flow provided simultaneously at the distal fibre tip (5 L/min). In comparison, a bipolar forceps was used at various power levels (20–60 W). Tissue coagulation and ablation were evaluated by white light images and vessel occlusion was visualised by optical coherence tomography (OCT) B-scans at a wavelength of 1060 nm. Coagulation efficiency was calculated by means of the quotient of the difference between the coagulation and ablation radius to the coagulation radius. Pulsed laser application achieved blood vessel occlusion rate of 92% at low pulse duration of 200 ms with no occurrence of ablation (coagulation efficiency 100%). Bipolar forceps showed an occlusion rate of 100%, however resulted in tissue ablation. Tissue ablation depth with laser application is limited to 40 μm and by a factor of 10 less traumatising than with bipolar forceps. Pulsed thulium laser radiation achieved blood vessel haemostasis up to 0.3 mm in diameter without tissue ablation and has proven to be a tissue-gentle method compared to bipolar forceps.

## Introduction

In the last decades, lasers in the infrared wavelength range were investigated for their potential as an instrument for neurosurgery. Considering the tissue-specific absorption of electromagnetic radiation as a function of wavelength, lasers with high water absorption seemed to be promising for precise and gentle procedures in brain tissue.

The first laser studies in neurosurgery investigated CO_2_ laser systems (wavelength at 10.6 µm), which are known for their precise cutting properties due to their high water absorption peak, but because of the destructive effect on blood vessels, conventional electrocautery instruments are continued to be used [1–3]. Nd:YAG laser systems (wavelength at 1.06 µm) showed better deep tissue coagulation due to its weak water absorption and were chosen in case of strong vascularization in brain tissue. The non-contact laser application offered a gentle procedure and reduction in tissue trauma in contrast to tissue touching bipolar forceps. But due to scattering effects and uncontrolled heating, unwanted tissue damage occurred in the surrounding tissue using Nd:YAG laser systems [4, 5]. Thulium laser emitting at 1940 nm wavelength with an absorption coefficient of 128 cm^−1^ [6, 7], which is less than the absorption coefficient of the CO_2_ laser (857.36 cm^−1^) but higher than of the Nd:YAG laser (0.6 cm^−1^) [8], offered a trade-off between limited penetration depth and sufficient coagulation property and became interesting for neurosurgery, in terms of ablation and coagulation for safe surgical tissue removal. The thulium laser has been successfully used in other medical fields for laparoscopy and minimally invasive surgery such as liver and kidney resection or in urological surgery [9–12]. Particularly existing studies showed great interest in the ablation properties (ablation efficiency) of the thulium laser, so various dosimetry studies were carried out to minimise the thermal effect and maximise the therapeutic effect of cutting, where different power, exposure time and laser modulation were tested in cortical, subcortical and tumour tissue. It provided the possibility of a tissue-gentle procedure in clinical application, e.g. neuroendoscopy without mechanical irritation, and proved to be time-saving, due to fewer instrument changes [13–15]. The photothermal effect differs in cortical and subcortical tissue since the water percentage is different and pulsed modulation caused lower ablation efficiencies [16, 17].

Laser application was tested beside conventional used ultrasonic aspirator and bipolar forceps for coagulation, tumour shrinking and resection. Though ultrasonic aspirator provided optimal tumour removal, it cannot coagulate, and a change of instrument to bipolar forceps is needed. On the other hand, thulium laser allowed the shrinkage and coagulation of tumour tissue avoiding surgical damage in the surrounding tissue and caused more defined procedure compared to bipolar forceps. Moreover, the thulium laser showed the ability to coagulate blood vessels (for submillimetre vessels), reducing intraoperative bleeding and minimising resection time [18–20].

Latest studies demonstrated optical coherence tomography (OCT)–guided thulium laser coagulation of microcirculation for an in vivo animal model and show that OCT-guided application enabled effective neural tumour tissue removal, whilst keeping the surgical field blood free [21, 22].

Since the ultrasonic aspirator showed a reliable performance in tumour tissue ablation and is used as a common instrument in neurosurgery, it should be discussed whether a combination of laser (haemostasis) and ultrasonic aspirator (tumour ablation) is suitable for tumour resection.

For this purpose, this work investigated the photocoagulation of cortical blood vessels in an ex vivo experiment on porcine brain tissue with a 1940-nm wavelength thulium laser system vs. conventional electrosurgical bipolar forceps. The objective of this study, contrasted with previous investigations, is to keep tissue ablation as low as possible in blood vessel coagulation. Of particular interest is the possibility of achieving a blood vessel occlusion by non-contact pulsed laser application. Thereby, a high coagulation efficiency is desired, avoiding the occurrence of tissue ablation.

## Materials and methods

**Fig. 1:**
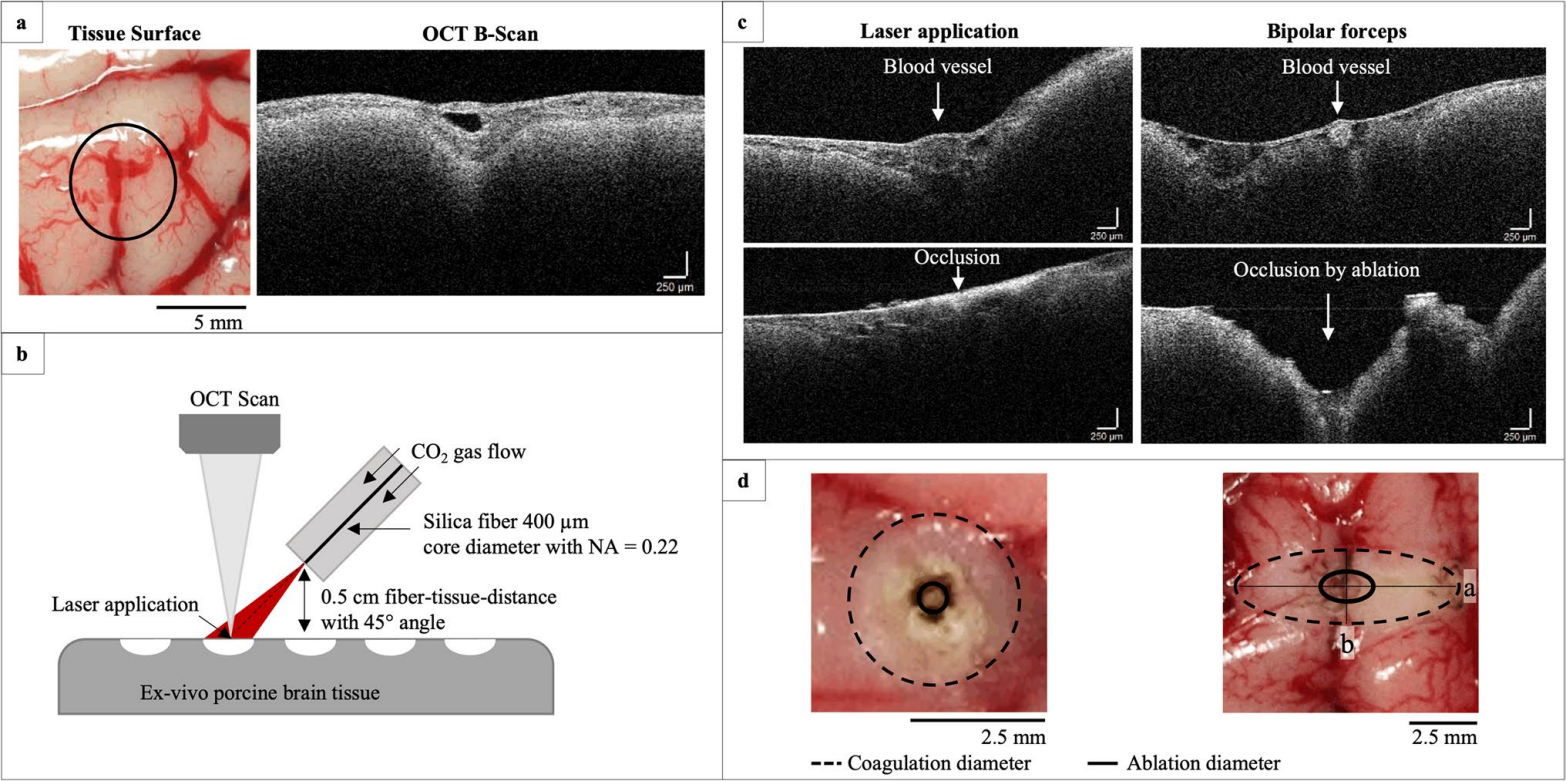
**a** Blood vessel on ex vivo porcine brain tissue surface and with OCT scan in cross-section. **b** Static thulium laser application with a tissue-fibre distance of 0.7 cm at 45°. A silica fibre with a core diameter of 400 µm and a NA = 0.22 was used with permanent or no CO_2_ gas flow respectively. Laser application and OCT scan were overlaid on the same spot. **c** Blood vessel occlusion by laser coagulation and bipolar forceps, demonstrated in OCT B-scan. **d** Evaluation of photo/-thermal tissue change area (coagulation and ablation)

Fresh ex vivo porcine brain tissue (slaughter material) was used within ~ 5 h after extraction. Ex vivo cortical blood vessels (on the surface) of porcine brain tissue (Fig. 1a) were irradiated with the Asclepion MultiPulse Tm + 1470 (Asclepion Laser Technologies GmbH, Jena, Germany) consisting of a high-power thulium and NIR module laser with the wavelengths of 1940 nm (thulium laser) and 1470 nm as well as pilot laser diode with a wavelength of 635 nm. Pulse mode was used with pulse durations of 100 to 500 ms (100-ms increments) at a repetition rate of 1 Hz and peak power of 15 W. Laser application lasted for 3 s. Laser light was guided through a fused silica bare fibre with a core diameter of 400 µm with a numerical aperture of 0.22 (Asclepion Laser Technologies GmbH, Jena, Germany) and applied in non-contact at an angle of 45° with a ~ 7-mm fibre-tissue distance (see Fig. 1b). Laser treatment took place at a room temperature of 21 °C. In addition, laser irradiation of the blood vessels was performed with CO_2_ gas flow (5 L/min) to observe a possible influence of the flow effect on the extent of photothermal tissue change. Furthermore, blood vessels were coagulated with bipolar forceps (VIO 300 D, Erbe Elektromedizin GmbH, Tübingen, Germany) with power of  20 W, 40 W and  60 W for 3 s.

For the evaluation of the blood vessel coagulation, pre- and post-images were captured with a white light camera (Canon Deutschland GmbH, Krefeld, Deutschland) provided with a macro-objective (EF100 mm f/2.8 MACRO USM, Ultrasonic). En face pictures were taken from laser and bipolar forceps treatment regarding coagulation and ablation area as well as of the tissue cross-section after treatment. Areas of photothermal/thermal tissue change were evaluated in ImageJ at the tissue surface as radii of coagulation (whitening of the tissue) and ablation (tissue destruction). For coagulation with bipolar forceps, two radii were evaluated (major *a* and minor *b*), as the thermal tissue change occurred elliptically, unlike the laser coagulation, where the damage was approximately circular area. Since no dominant black carbonisation was found in this study, initial crusting/dehydrating of the tissue was also classified as coagulation (see Fig. 1d). The same kind of photothermal/thermal tissue changes was evaluated in the tissue depth. OCT images with a spectral domain OCT at a wavelength of ~ 1060 nm were captured (Thorlabs GmbH, Dachau, Deutschland) which enabled the selection of blood vessels to be treated.

To ensure OCT imaging took place at the same position as the laser treatment, the OCT scan line was overlaid on the pilot laser of the thulium laser. Images were taken before and after laser and bipolar forceps treatment to investigate photo/-thermal tissue changes. In addition, OCT imaging enabled the assessment whether laser irradiation was able to achieve blood vessel occlusion and whether these coagulations included ablative zones (Fig. 1c). In OCT, coagulation on the tissue surface could not be distinguished, which is why only ablation was considered on the tissue surface as well as in the tissue depth. Because the ablation in the tissue cross-section was not clearly visible in the white light images, only the coagulation depth was considered here and combined with the observed ablation (if there were any) from the OCT images. Coagulation efficiency was calculated by the quotient of coagulation and ablation:

1 $$\frac{\mathrm{Coagulation\; radius}-\mathrm{Ablation\; radius}}{\mathrm{Coagulation\; radius}}*100$$

## Results

After laser application on ex vivo porcine cortical brain blood vessels, laser-induced thermal tissue change was classified into desirable coagulation and non-desirable ablation at tissue surface and depth by white light and OCT images. Thereby, parameters could be selected resulting in a defined laser coagulation and achieving occlusion of the blood vessels. In addition, coagulation efficiencies for chosen parameters were determined. Furthermore, tissue change and blood vessel occlusion behaviour after coagulation were compared to bipolar forceps application.

### Appearance of thermal tissue alteration by laser and bipolar forceps

**Fig. 2:**
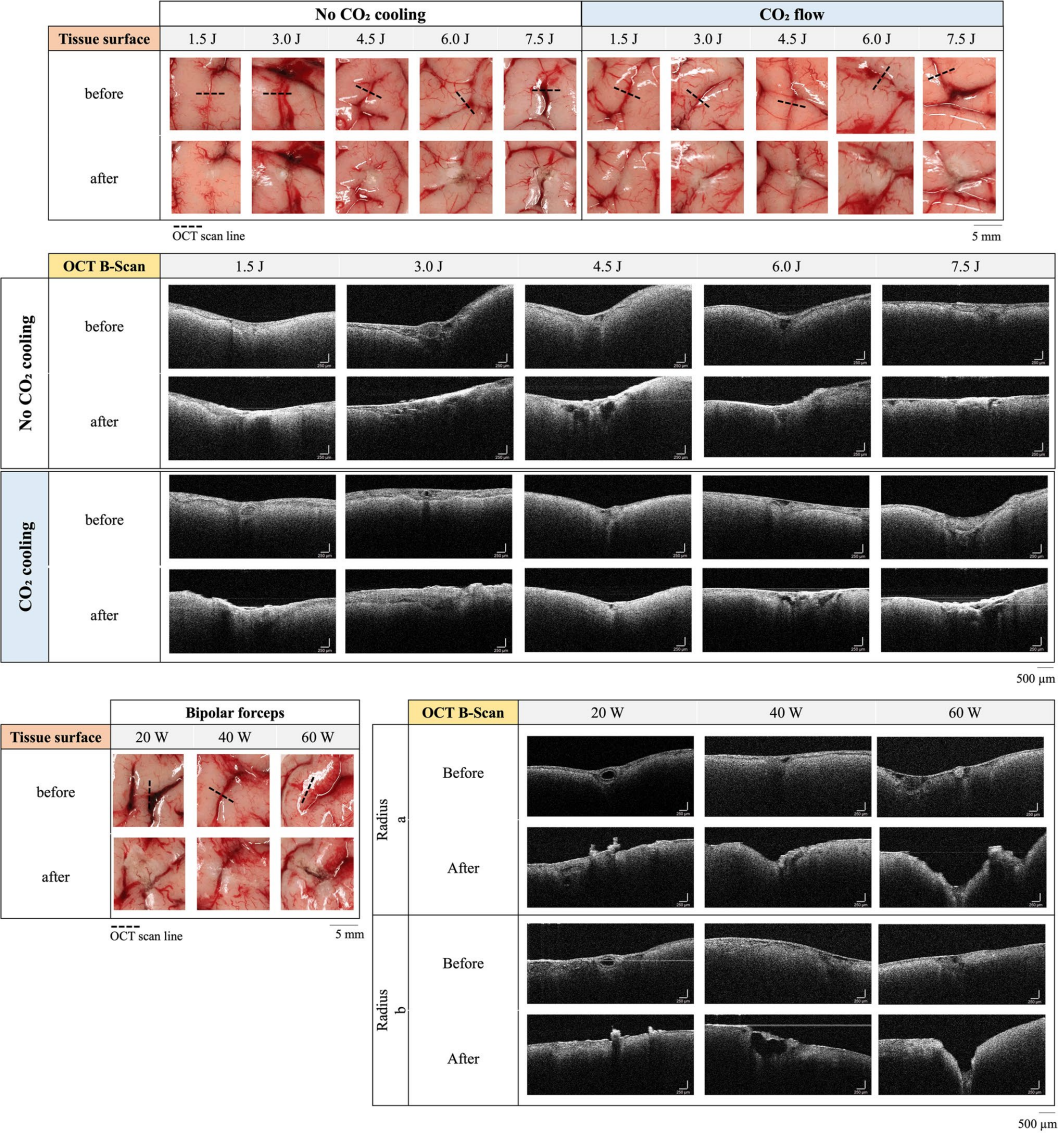
Macroscopic images and OCT B-scan before and after thulium laser application with and without CO_2_ gas flow (top) and bipolar forceps (bottom). An increase of the pulse energy resulted in an increase of the photothermal tissue change. OCT B-scans show the blood vessel in cross-section and enabled the assessment of blood vessel occlusion and occurrence of ablation

White light images in Fig. 2 show blood vessels before and after static laser application with 1940 nm with and without CO_2_ gas flow. After laser application, a photothermal tissue change of the blood vessel is obtained in the form of whitening and crusting of the tissue for chosen laser parameter. The laser induces a defined photothermal tissue change (coagulation, ablation) in an approximately circular area. For white light images, no ablation was observed.

The longer the pulse duration from 100 to 500 ms (pulse energy 1.5 to 7.5 J), the greater the photothermal tissue change on the tissue surface.

With CO_2_ gas flow, laser-induced tissue change seems to increase at a slower rate. OCT B-scan (Fig. 2) shows the cross-section of the arachnoid and the blood vessels within, which can be clearly distinguished here from the cerebral matter beneath. Photothermal tissue change can be detected as an increased scattered signal in the B-scan. Blood occlusions due to laser application can be imaged with OCT.

Due to the photothermal impact, an increased scattering signal appears in the treated area, where an alteration of the tissue surface (arachnoid) can be observed and in case of blood vessel occlusion of the original structure of the blood vessel is no longer intact.

Conventional application with bipolar forceps produced elliptical tissue damage at tissue surface; therefore, it was evaluated in two damage radii. Occurring ablations due to coagulation with the bipolar forceps are clearly detected in the OCT (see Fig. 2, e.g. at 60 W).

### Coagulation and ablation radii, coagulation efficiencies and blood vessel occlusion rate

**Fig. 3:**
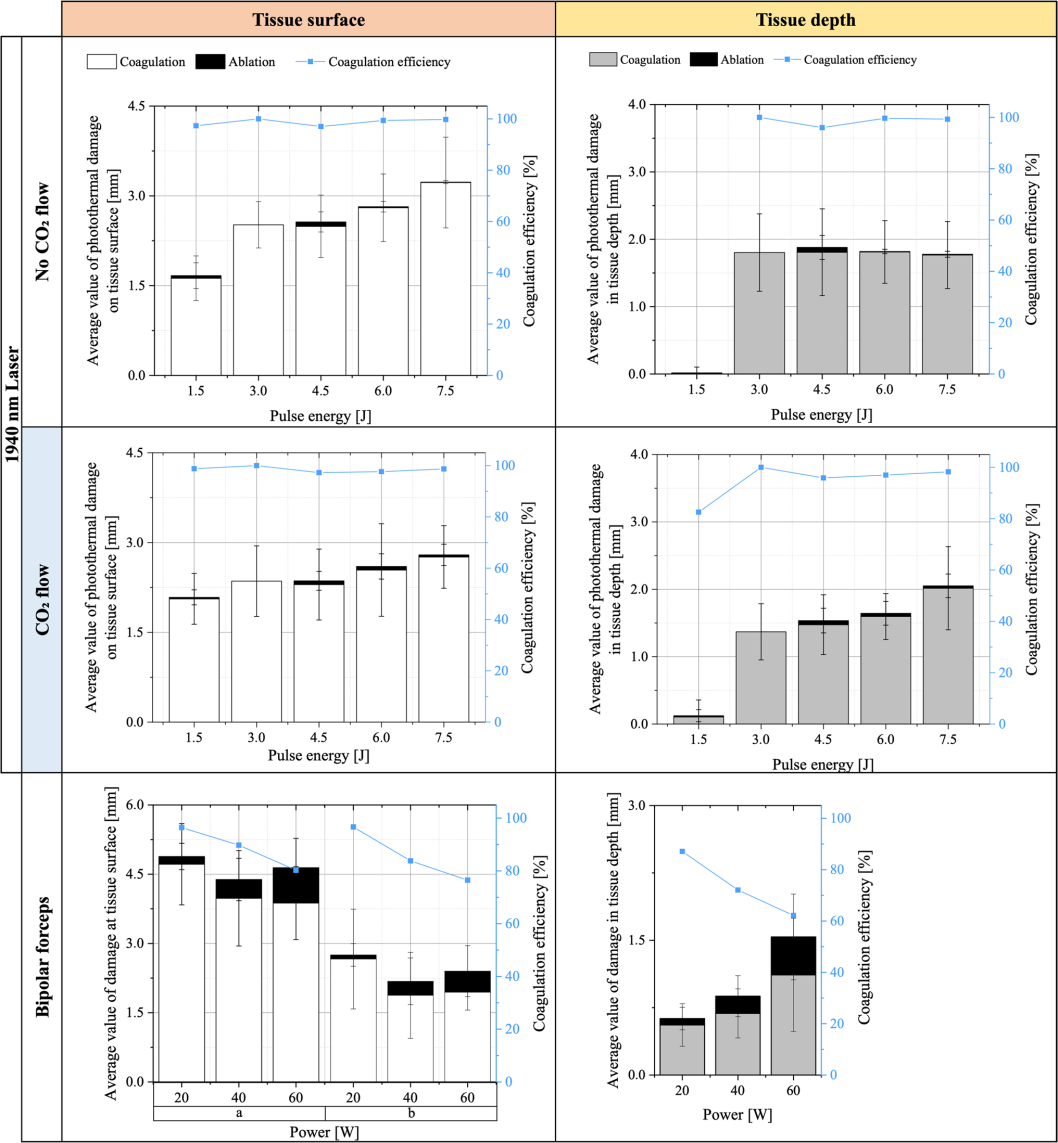
Average values of the photothermal tissue change as radius at tissue surface and depth after pulsed thulium laser irradiation with and without CO_2_ flow. Coagulation and ablation are evaluated for different pulse energies (1.5 to 7.5 J). Photothermal tissue change is compared to thermal change by bipolar forceps with power of 20 to 60 W (used in clinical application). Coagulation efficiencies are shown as function of pulse energy or power

Laser-induced photothermal tissue changes at tissue surface are demonstrated in Fig. 3 as average value of coagulation and ablation radius as a function of applied pulse energies 1.5 to 7.5 J. For each laser parameter, a total of 5 samples were treated, irradiating 5 blood vessels each, so that a total of 25 blood vessels were treated and examined.

Laser application without CO_2_ gas flow shows a photothermal tissue change with a high percentage of coagulation. Minimal coagulation was found at the lowest pulse energy of 1.5 J with an average radius of 1.3 mm, with a 0.04 mm radius ablation at the centre.

Pulse energy increase led to a linear increase in photothermal tissue change, where highest pulse energy of 7.5 J reached a total damage radius of 3.23 mm, with the largest fraction of coagulation of 3.22 mm.

Maximum tissue ablation by laser application without CO_2_ gas flow of 0.07 mm was achieved with a pulse energy of 4.5 J. Coagulation only was reached at 3 J, with radius of 2.5 mm.

Laser application with CO_2_ flow led to a less rapid increase in photothermal tissue change size with increasing pulse energy. With the maximum pulsed energy of 7.5 J, a reduced total photothermal tissue change of 2.80 mm radius was achieved compared to laser irradiation without CO_2_ gas flow. Contrasted to the irradiation with CO_2_ gas flow, laser application at the lowest pulse energy produced a larger photothermal tissue change with a coagulation radius of 2.07 mm, and 0.03 mm ablation radius. The total damage for laser application with CO_2_ gas flow remained lower for pulse energies from 3 to 7.5 J compared to irradiation without CO_2_ gas flow effect. Results of ablation radii were not significantly different from laser application without CO_2_ gas flow. A maximum tissue ablation of 0.06 mm radius was achieved on the tissue surface at 4.5 J and 6.0 J pulse energy.

At the lowest selected power of 20 W, the maximal thermal tissue change was achieved with 4.8 mm for the major radius, which is double the size of laser-induced photothermal tissue change. With increasing power of up to 60 W, the thermal tissue change decreased for both radii at tissue surface. The ablation radius increased with increasing power from 0.17 mm (20 W) to 0.77 mm (60 W) on the tissue surface, which at the highest reading power setting of 60 W is 10 times greater than with laser application.

Laser-induced tissue change in depth showed a sharp slope from 1.5 to 3 J pulse energies and remained steady up to 7.5 J for application with CO_2_ gas flow, whilst no CO_2_ gas flow induced a linear increase of tissue change in depth with rising pulse energy. Total photothermal tissue change in depth is limited to 1.8 mm using no CO_2_ gas flow, whilst with CO_2_ gas flow a greater damage depth of 2.0 mm was reached. The highest ablation in depth by laser application occurred at 4.5 J with 0.07 mm. Remarkable was the sharp increase of photothermal tissue change in tissue depth from 1.5 to 3 J in both laser application cases and the highest ablation was observed at 4.5 J with 0.07 mm.

Application with bipolar forceps resulted in an ablation depth of 0.07 mm at the lowest power of 20 W. Coagulation depth at the lowest applied power was 0.6 mm. With increasing power, the total thermal tissue change in the tissue depth increased and with it the ratio of ablation. At the maximum selected power of 60 W, the thermal tissue change in depth of 1.5 mm is less than with laser application; however, the occurring ablation of 0.4 mm had a much greater destructive effect.

Coagulation efficiency of at least 95% was achieved by laser application with and without CO_2_ gas flow for selected pulse energies (see Table 1). Complete coagulation efficiency (100%) was found for treatment with 3 J pulse energy in both laser application cases for tissue surface and depth. When compared, the bipolar forceps achieves a maximum coagulation efficiency of 97% at the lowest power setting of 20 W. At tissue depth, the coagulation efficiency is only 87%. The highest occlusion rate of blood vessels during laser application with CO_2_ gas flow was realised at 1.5 J and 3 J with 92%. No CO_2_ gas flow resulted in a much lower occlusion rate of less than 90%.

**Table 1:** Coagulation efficiencies and blood vessel occlusion rate in % at tissue surface and depth for pulsed thulium laser application with pulse energies of 1.5 to 7.5 J compared with bipolar forceps

Laser 1940 nm							Bipolar forceps			
	No CO2 cooling			CO2 cooling						
Pulse energy (µJ)	Coagulation efficiency [%]		Blood vessel occlusion rate [%]	Coagulation efficiency [%]		Blood vessel occlusion rate [%]	Power [W]	Coagulation efficiency [%]		Blood vessel occlusion rate [%]
	S	D		S	D			S	D	
1.5	97	0	64	99	83	92	20	97	87	89
3	100	100	84	100	100	92	40	84	72	100
4.5	97	96	88	97	96	88	60	76	62	100
6	99	100	88	98	97	84				
7	100	99	80	99	98	88				

## Discussion

In neurosurgery, high-risk functional tissue is treated, making it necessary to use very tissue-gentle instruments for neurosurgical procedures such as blood vessel coagulation (haemostasis). Furthermore, the surgical field should remain clear and the instruments should make it more convenient for the surgeon to operate. Until now, neurosurgery has used the ultrasonic aspirator and bipolar forceps for tumour removal and haemostasis, but this requires a change of instruments. Also, during vascular coagulation with bipolar forceps, the in-contact procedure results in mechanical tissue destruction from sticking and carbonisation.

A combination of laser coagulator and ultrasonic aspirator would combine the advantages of sufficient ablation property and gentle, non-contact coagulation and eliminate the need to change instruments, thus providing a gentle and time-saving procedure alternative.

The heat generation in tissue and the level of tissue damage depend on factors such as on the irradiation time, laser energy and operation mode (CW or pulsed), as well as the wavelength-specific absorption coefficient in the target tissue. Since biological tissue is largely composed of water [20, 23], lasers of the infrared wavelength range with a high water absorption rate have been studied for neurosurgical laser application in brain tissue. Coagulation light is converted into heat, leading to tissue temperatures in the range of 60 to 100 °C. When laser is applied to blood vessels, the coagulation leads to the denaturation of proteins and collagen in the vascular wall, which is visible as macroscopic whitening of the tissue [24–26]. In case of blood flow, heating might change due to the cooling effect of the blood flow, and parameter for coagulation must be adjusted.

Reasons why lasers have not been established yet in neurosurgery are uncontrolled tissue interactions due to unsuitable laser wavelengths, which lead to either photoevaporative effect and insufficient coagulation (CO_2_ Laser) [2] or deep tissue penetration depth (ND:YAG laser) [4] and related injury of vessels [3]. Recent study showed the potential of the thulium laser, because of its high water absorption peak at 2 µm wavelength [6, 7] and resulting limited penetration depth. The possibility of guiding the laser beam through a flexible silica fibre makes the thulium laser suitable for microsurgery and endoscopy. The thulium laser was used preliminary in neurosurgical studies with high interest in ablation efficiency [13, 15] and has been proven to be beneficial for neuroendoscopy, tumour shrinkage and resection and application near nervous structures [12–14, 18–20]. Compared to ultrasonic aspirator, the thulium laser shows the additional ability to coagulate small vessels in tumour without the requirement of the bipolar cautery, but still ultrasonic aspirator has the best ablation performance [19]. Different from bipolar forceps, which cause irregular thermal damage, the thulium laser induces coagulation more precisely [18]. Compared to irregular thermal changes by bipolar forceps, the thulium laser in this study showed a nearly circular coagulation area. By laser irradiation the tissue heats up due to scattering and absorption. The longer this irradiation lasted, the warmer it became inside the tissue and the greater the thermal expansion. The coagulation could spread due to the thermal expansion. An increase of the photothermal tissue change by longer irradiation could be shown in this study by increasing pulse lengths (100 to 500 ms), whilst the irradiation duration (3 s) remained the same. Absorption properties are changed in the treated area and the frequent irradiation leads to stronger heating of the tissue, which may support the occurrence of ablation. In case of a gas flow effect, the thermal expansion is reduced and tissue alterations are kept locally. The use of CO_2_ gas flow led to a slower increase of photothermal tissue change at tissue surface, reducing the heat distribution. Whilst the photothermal tissue change in tissue depth was reduced by CO_2_ gas flow which only increases linearly to higher pulse energies, the highest coagulation depth with no CO_2_ gas flow is reached at low pulse energy and remains the same. Used CO_2_ gas flow fibre provides a flow effect of the tissue and on top is useful in case of bleeding, to keep the operation field free.

The objective of this study, contrasted with previous investigations, is to keep ablation as low as possible in static irradiation, with a view to clinical application for blood vessel coagulation and to achieve a limited thermal damage in blood vessel dimension. To achieve high coagulation efficiencies, for blood vessel coagulation, pulsed laser irradiations with 100 to 500 ms pulse duration were found to be adequate and the blood vessel occlusion could be observed in OCT B-scans. Due to laser pulsing with a low repetition rate of 1 Hz in our study, the tissue is able to cool itself during heating phases and the thermal damage is locally controlled. An increase in pulse energy (1.5 to 7.5 J) leads to a linear rise in thermal damage at the surface. Thereby, the percentage of coagulation is dominant and in case of ablation damage was limited to a submillimetre range. Although the selected pulse energy of up to 7.5 J was higher compared to previous studies (4 J [13, 14]), a low ablation respectively high coagulation efficiency of up to 100% was achieved. However, the total thermal damage depth was higher with thulium laser than with bipolar forceps, and the fraction of ablation is less by a factor of 10. Coagulation depth of < 2 mm seemed to be appropriate since treated blood vessels show a diameter of 350 µm. The highest occlusion rate with minimal destructive coagulation can be obtained with the thulium laser with CO_2_ gas flow for 3 J pulse energy, where a blood vessel occlusion rate of > 90% was found with a coagulation efficiency of 100%, whilst for bipolar forceps complete occlusion goes along with ablation behaviour (< 85% coagulation efficiency). The non-contact application of the thulium laser fibre avoided mechanical trauma of the brain tissue and vessel. The angled positioning of the laser fibre simulated the guidance of the fibre by the neurosurgeon and the flexibly of the laser fibre makes a combination with other instruments possible. Since the thermal effect of unfocused laser only occurs at a limited distance, the surgeon must be trained before applying the laser in the operating field. If the surgeon is placing the fibre to close to the tissue, carbonisation or even ablation can occur earlier, with the same parameter settings tested in this work at selected distance.

**Fig. 4:**
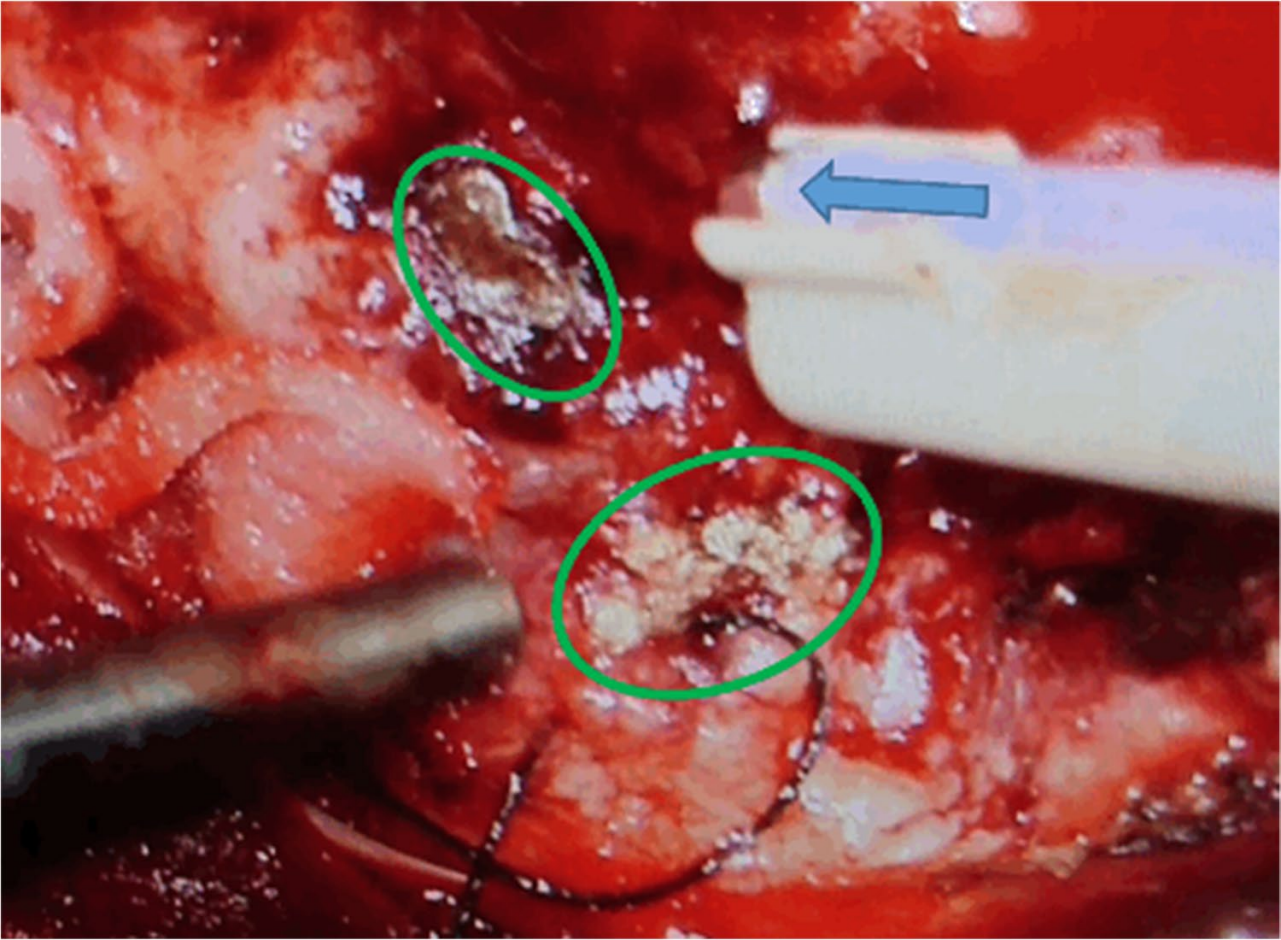
In vivo clinical use of thulium laser system during meningioma resection. Blue arrow: Laser fibre embedded in a gas flow tube; arrow points into emission direction of laser beam (power 15W, pulse duration 400 ms, repetition rate 1 Hz). Green marked areas: Coagulated tissue for haemostasis. Suction instrument on the left was used to point to the already coagulated areas

Since June 2022, the system is evaluated within a clinical study on brain tumour resection approved by the ethics committee of the University of Lübeck [No. 19–319]. All patients have confirmed their participation in written consent. Initial results are very promising and the operating clinicians very much appreciate the ease of use of the system. Figure 4 shows the thulium laser application during a tumour resection. Comprehensive results of the study will be published after its completion.

## Conclusion

Pulsed thulium laser at 1940 nm together with CO_2_ gas flow achieved non-ablative blood vessel haemostasis and has proven to be a tissue-gentle method compared to bipolar forceps. Future plans include integrating the flexible laser fibre (0.7 mm in diameter) into an ultrasonic aspirator system for intraoperative direct coagulation of bleeding during ultrasonic aspiration without interrupting the intraoperative workflow. The results of the ongoing clinical trial need to be evaluated to validate the concept of small vessel haemostasis by thulium laser.
